# Supplementary material for: High microphone signal-to-noise ratio enhances acoustic sampling of wildlife
Source: PeerJ. 2020 Oct 20;8:e9955. doi: 10.7717/peerj.9955 (PMC7585376; doi:10.7717/peerj.9955)
Supplement: Supplemental Information 1 [file peerj-08-9955-s001.docx]

# Supplementary Materials

Title: Microphone signal-to-noise ratio affects acoustic sampling of wildlife

Authors: Kevin Darras, Franziska Deppe, Yvonne Fabian, Agus Priyono Kartono, Andres Angulo Rubiano, Yeni A. Mulyani, Dewi Prawiradilaga

## Microphone assembly

To construct microphones, wires were soldered onto the microphone element contacts and the wires were connected to Mini-Con-X audio connectors (Part number: 6282-3PG-3DC, Conxall/Switchcraft) so that they could be plugged into our automated sound recorders (Song Meter SM2Bat+, Wildlife acoustics). All capsule microphones and one older generation MEMS microphone needed a 2.5 V bias to operate, which can be enabled inside the recorder via jumper switches. We needed to record sounds at similar levels, so we amplified the microphones' outputs according to the sensitivity specification of the manufacturer. We used a reference amplification value of 48 dB, which is the recommendation of the recorder manufacturer for its SMX-II microphone, which has a sensitivity of -35 dB. Even so, the POM-1345P-C3310-R microphone outputs very low sound levels so that we increased the amplification (by 60 dB).

It appears that MEMS microphones are more efficient at recording ultrasound, maybe because of the smaller size of the microphone diaphragm, which can respond more quickly to high-frequency air oscillations.
