# Supplementary figures and images for: High microphone signal-to-noise ratio enhances acoustic sampling of wildlife
Source: PeerJ. 2020 Oct 20;8:e9955. doi: 10.7717/peerj.9955 (PMC7585376; doi:10.7717/peerj.9955)

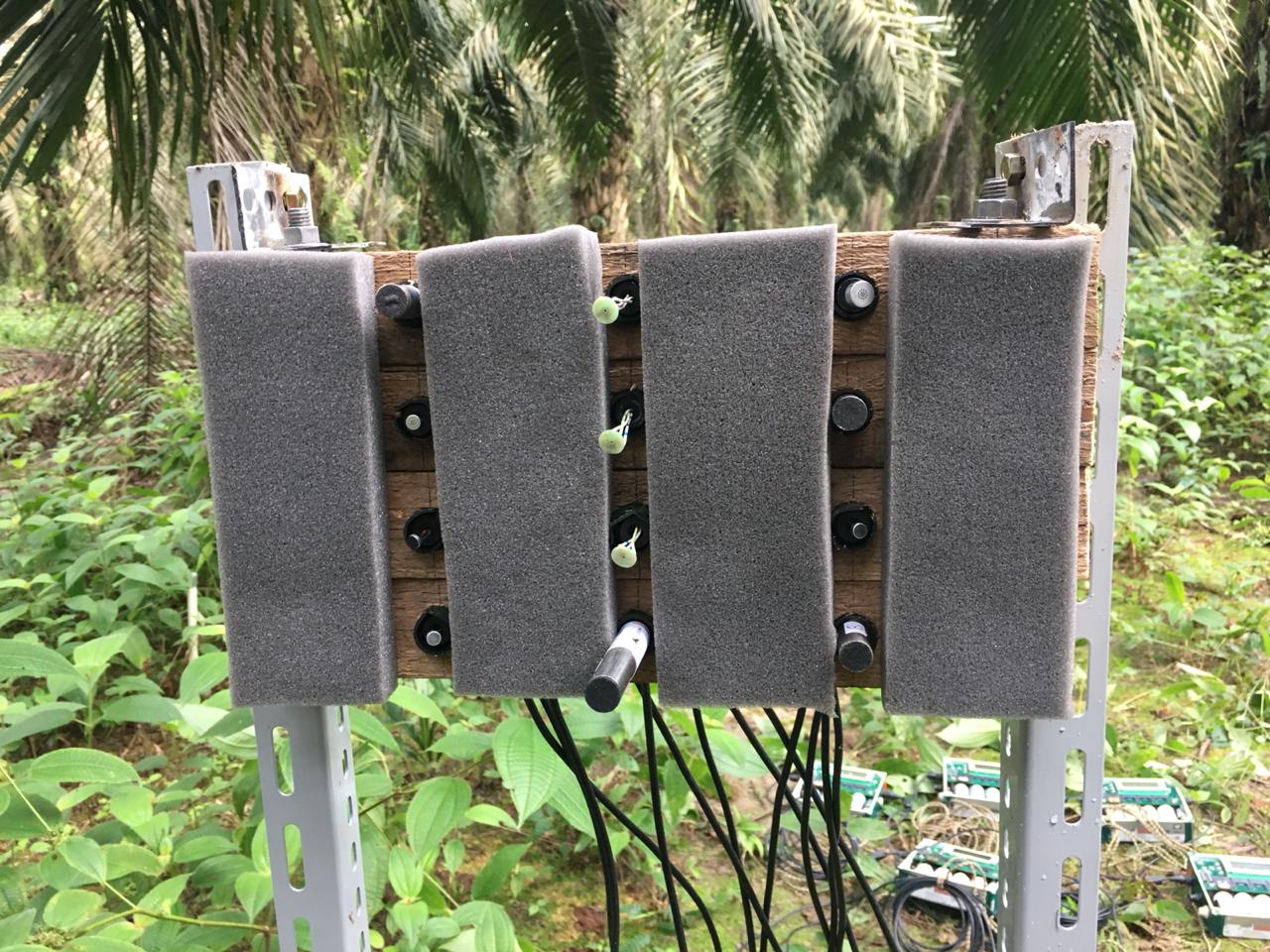

Supplement: Figure S1 — The wooden pane, approximately 25 cm × 35 cm, was installed at a height of 1.5 m and padded with foam, to avoid friction noise. The microphones are spaced 10 cm vertically and 15 cm horizontally. [file peerj-08-9955-s002.jpeg]

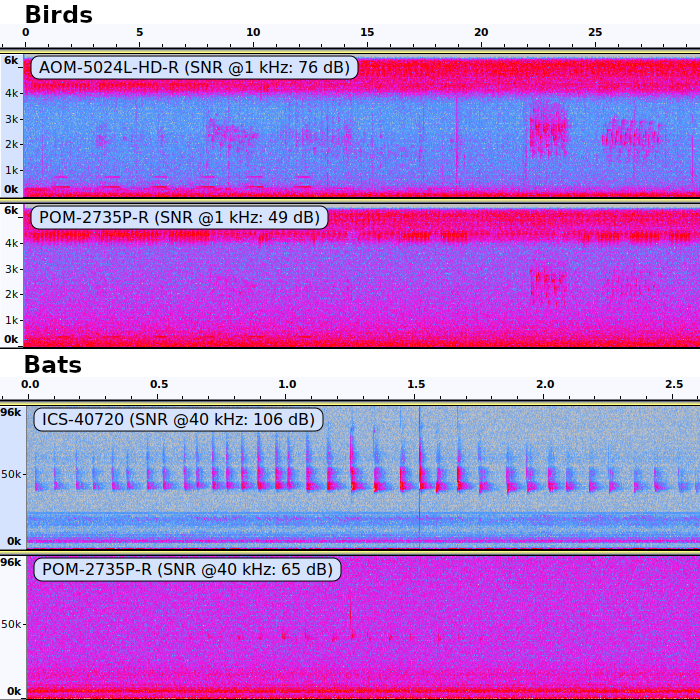

Supplement: Figure S2 — Microphone models mentioned in black font, the recorder was a SM2Bat+ (Wildlife Acoustics, Massachusetts, USA). Spectrograms generated using Audacity with a Hanning window size of 1024, no gain. Time on the X axis (MM:SS format) and frequency on the Y axis (kHz). [file peerj-08-9955-s003.png]

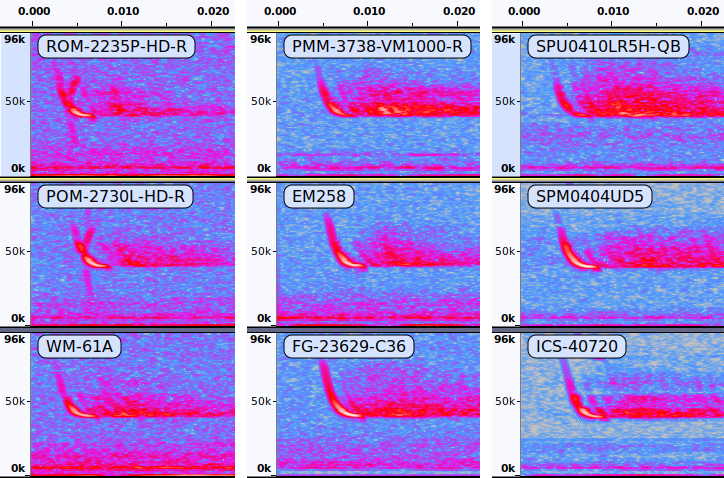

Supplement: Figure S5 — Microphone models indicated in overlaid text, time in seconds on the X axis, frequency in kHz on the Y axis, amplitude shown with a scale from blue (low) to red (high) and white (highest). All spectrograms were equalised to peak amplitude, ordered by signal-to-noise ratio (from top to bottom, then left to right) and captured from Audacity with a FFT window size of 256. [file peerj-08-9955-s006.png]
