# Supplementary material for: High microphone signal-to-noise ratio enhances acoustic sampling of wildlife
Source: PeerJ. 2020 Oct 20;8:e9955. doi: 10.7717/peerj.9955 (PMC7585376; doi:10.7717/peerj.9955)
Supplement: Supplemental Information 3 [file peerj-08-9955-s008.html]

Microphone Signal-to-noise Ratio Analysis


# Microphone Signal-to-noise Ratio Analysis

#### Kevin FA Darras

#### June 26, 2020

# Preparation

clear all variables, load packages

```
rm(list=ls())

library(MASS)
library(data.table)
library(ggplot2)
library(cowplot)
```

```
## 
## ********************************************************
```

```
## Note: As of version 1.0.0, cowplot does not change the
```

```
##   default ggplot2 theme anymore. To recover the previous
```

```
##   behavior, execute:
##   theme_set(theme_cowplot())
```

```
## ********************************************************
```

```
library(monitoR)
```

```
## Loading required package: tuneR
```

```
## 
## Attaching package: 'monitoR'
```

```
## The following object is masked from 'package:tuneR':
## 
##     readMP3
```

```
library(DHARMa)
```

```
## This is DHARMa 0.3.1. For overview type '?DHARMa'. For recent changes, type news(package = 'DHARMa') Note: Syntax of plotResiduals has changed in 0.3.0, see ?plotResiduals for details
```

```
library(MuMIn)
library(emmeans)
```

set working directory

```
setwd("/home/kdarras/DATA/Docs/Boulot/2012_Jambi/Microphone SNR")
```

import data from CSVs

```
types0=fread("Microphones - Types.csv")
mics0=fread("Microphones - Units.csv")
extinction0<-fread("Microphones - Extinction distances.csv")
tags0=fread("Bird and bat tags.csv")
```

derive tag duration and microphone IDs

```
tags0[,duration_s:=(max_time-min_time)]
tags0[,Microphone_ID:=as.numeric(substr(recording.name,1,2))]
```

check unique species (empty strings correspond to recordings with no tags)

```
tags0[,unique(binomial)]
```

```
##  [1] ""                       "Chalcophaps indica"     "Dicaeum trigonostigma" 
##  [4] "Geopelia striata"       "Halcyon smyrnensis"     "Orthotomus atrogularis"
##  [7] "Orthotomus ruficeps"    "Orthotomus sericeus"    "Pycnonotus aurigaster" 
## [10] "Pycnonotus goiavier"    "A1"                     "B1"                    
## [13] "C1"                     "D1"
```

Calculate electrical noise floor of microphones to compare to
recorder pre-amplifier noise contribution. Do not calculate for SMX-US1
because of built-in amplification. Pre-amp noise floor is -105 dBV at
192 kHz sampling rate according to Wildlife Acoustics specifications

```
types0[Code!="K",electrical_noise_floor_dBV:=(Sensitivity_specified_dBV-SNR_specified_1kHz_dB)]
```

merge microphones with types info

```
mics1=merge(mics0,types0,by="Code")
```

# Microphone SNR

standardise self-noise and sound levels at closest distance by subtracting amplification of recorder

```
mics1[,c("selfnoise_1kHz_dB_noamp","selfnoise_40kHz_dB_noamp","soundlevel_1kHz_4m_level1_dB_noamp","soundlevel_40kHz_8m_dB_noamp"):=
        .(selfnoise_1kHz_dB-Amplification_recorder_dB
          ,selfnoise_40kHz_dB-Amplification_recorder_dB
          ,soundlevel_1kHz_4m_level1_dB-Amplification_recorder_dB
          ,soundlevel_40kHz_8m_dB-Amplification_recorder_dB)]
```

further subtraction for FG Knowles microphone (Type F) that has built-in amplification of 48 dB

```
mics1[Code=="F",c("selfnoise_40kHz_dB_noamp","soundlevel_40kHz_8m_dB_noamp"):=
        .(selfnoise_40kHz_dB_noamp-48,soundlevel_40kHz_8m_dB_noamp-48)]
```

get calibration value at 1 kHz for reference microphone (ID 9;
amplification was 36dB for calibration) TODO: use calibration value for
each set?

```
calibration_value_1kHz_dB=mics1[Microphone_ID==9,soundlevel_94dBSPL_1kHz]-36
```

The US calibrator’s “CAL” mode emits a 48 dB SPL (+/- 3dB) 40 kHz
signal at a distance of 30 cm according to Wildlife acoustics
documentation

```
calibration_value_40kHz_dB=mics1[Microphone_ID==15,soundlevel_48dBSPL_40kHz]-36
```

calculate sensitivities of all microphones relative to reference microphone

```
mics1[,relative_to_reference_1kHz_dB:=soundlevel_1kHz_4m_level1_dB_noamp-mics1[Microphone_ID==9,soundlevel_1kHz_4m_level1_dB_noamp]]
mics1[,relative_to_reference_40kHz_dB:=soundlevel_40kHz_8m_dB_noamp-mics1[Microphone_ID==15,soundlevel_40kHz_8m_dB_noamp]]
```

calculate SNR using calibration value, relative sensitivities, and self-noise

```
mics1[,SNR_1kHz_dB_cal:=calibration_value_1kHz_dB+relative_to_reference_1kHz_dB-selfnoise_1kHz_dB_noamp]
mics1[,SNR_40kHz_dB_cal:=calibration_value_40kHz_dB+relative_to_reference_40kHz_dB-selfnoise_40kHz_dB_noamp]
```

adjust SNR with loss of dB caused by acoustic vent (only used later in graphical comparison to manufacturer-provided values)

```
mics1[,SNR_1kHz_dB_cal_adjusted:=SNR_1kHz_dB_cal+Acoustic_vent_loss_dB]
```

We expect a linear relationship between specified and measured SNR and test different models.

Tested (likely) model: simple (constant) offsets in SNR values between manufacturers

```
lm.SNR=lm(SNR_1kHz_dB_cal_adjusted~SNR_specified_1kHz_dB+Manufacturer-1,mics1)
```

Complex model: offsets in SNR values between manufacturers and also differing slopes

```
lm.SNR.full=lm(SNR_1kHz_dB_cal_adjusted~SNR_specified_1kHz_dB*Manufacturer,mics1)
```

Simple model: no differences between manufacturers

```
lm.SNR.simple=lm(SNR_1kHz_dB_cal_adjusted~SNR_specified_1kHz_dB,mics1)
```

Null (unlikely) model: no linear relationship, only effect of manufacturer

```
lm.SNR.manu=lm(SNR_1kHz_dB_cal_adjusted~Manufacturer,mics1)
```

since models differ in number of predictors, we cannot use R-squared
measures for comparison. So we determine best model with information
criterion to counteract automatic positive effect of number of
predictors on fit.

```
AICc(lm.SNR,lm.SNR.full,lm.SNR.simple,lm.SNR.manu)
```

```
##               df     AICc
## lm.SNR         8 157.9336
## lm.SNR.full    9 163.8536
## lm.SNR.simple  3 175.8383
## lm.SNR.manu    7 190.4122
```

We find that the tested model with constant offsets for each
manufacturer is the most parsimonious. We can check model diagnostics
even though we have a fairly robust understanding of the relationships
so that it should be correct

```
plot(simulateResiduals(lm.SNR))
```

no striking problem.

We check its summary (one coult test for significant differences between manufacturers, but this is not the aim).

```
summary(lm.SNR)
```

```
## 
## Call:
## lm(formula = SNR_1kHz_dB_cal_adjusted ~ SNR_specified_1kHz_dB + 
##     Manufacturer - 1, data = mics1)
## 
## Residuals:
##      Min       1Q   Median       3Q      Max 
## -11.1835  -1.8225  -0.0864   1.3231  13.2987 
## 
## Coefficients:
##                        Estimate Std. Error t value Pr(>|t|)    
## SNR_specified_1kHz_dB    1.6296     0.1981   8.228 6.09e-07 ***
## ManufacturerInvensense -48.2839    14.4452  -3.343 0.004453 ** 
## ManufacturerKnowles    -30.3554    12.4174  -2.445 0.027330 *  
## ManufacturerPanasonic  -32.1375    12.9321  -2.485 0.025237 *  
## ManufacturerPrimo      -45.3422    15.2072  -2.982 0.009315 ** 
## ManufacturerPUI Audio  -56.5021    13.4720  -4.194 0.000782 ***
## ManufacturerVesper     -38.8375    12.9321  -3.003 0.008915 ** 
## ---
## Signif. codes:  0 '***' 0.001 '**' 0.01 '*' 0.05 '.' 0.1 ' ' 1
## 
## Residual standard error: 5.735 on 15 degrees of freedom
##   (2 observations deleted due to missingness)
## Multiple R-squared:  0.9944, Adjusted R-squared:  0.9917 
## F-statistic: 377.7 on 7 and 15 DF,  p-value: 1.05e-15
```

The low P-value for the SNR\_specified\_1kHz\_dB predictor tells us that
when assuming the null hypothesis (that this coefficient is 0) is true,
it is highly unlikely to get values at least as extreme as the one we
estimated.

Now we extract model predictions for plotting them subsequently.

```
predict.SNR=predict(lm.SNR)
```

We append these predictions to the microphones table.

```
mics2=cbind(mics1[as.numeric(names(predict.SNR))],SNR_predicted=predict.SNR)
```

We graph specified vs. measured SNR values along with the best
model’s fit, we use the calibrated SNR values (corrected for acoustic
vent presence).

```
ggplot(mics2,aes(SNR_specified_1kHz_dB,SNR_1kHz_dB_cal_adjusted,color=Manufacturer,label=Code,shape=Format))+
  geom_point()+
  geom_text(data=mics1[label_placement==1],nudge_y=2,show.legend=F)+
  geom_line(aes(SNR_specified_1kHz_dB,SNR_predicted),size=1)+
  scale_color_brewer(type="qual",palette=6)+
  xlim(c(20,80))+ylim(c(20,80))+
  geom_abline(slope = 1,intercept = 0,lty=3)+
  labs(x="Signal-to-noise ratio at 1 kHz\n(dB, manufacturer-given)",y="Signal-to-noise ratio at 1 kHz\n(dB, measured)")+
  theme_cowplot()+
  theme(legend.position = c(0.1,0.7))
```

```
ggsave("Figures/Fig S4.eps",width=5,height=5)
```

We explore graphically whether measured SNR at 1 kHz correlates with
SNR ar 40 kHz (we exclude the Knowles FG microphone that does not record
audible sound and thus has no SNR at 1 kHz).

```
ggplot(mics1[Code!="K"],aes(SNR_1kHz_dB_cal_adjusted,SNR_40kHz_dB_cal,color=Manufacturer,label=Code,shape=Format,group=1))+
  labs(x="Signal-to-noise ratio at 1 kHz\n(dB, measured)",y="Signal-to-noise ratio at 40 kHz\n(dB, measured)")+
  # stat_smooth(method="lm",se=T,color="black")+
  geom_point()+
  scale_color_brewer(type="qual",palette=6)+
  geom_text(nudge_y = 2)+
  # xlim(c(10,80))+ylim(c(10,80))+
  # geom_abline(slope=1,intercept = 0,lty=3)+
  annotate(geom="text",x=50,y=100,label=paste("Correlation:\n",
    round(cor(mics1[!is.na(SNR_1kHz_dB_cal),SNR_1kHz_dB_cal],mics1[!is.na(SNR_1kHz_dB_cal),SNR_40kHz_dB_cal]),2)))+
  theme_cowplot()+
  theme(legend.position = c(0.1,0.7))
```

```
ggsave("Figures/Fig S4.eps",width=5,height=5)
```

We select microphones with extreme SNR values for generating Fig S5.

```
mics1[set==1,SNR_40kHz_dB_cal,.(Model,Microphone_ID)]
```

```
##                 Model Microphone_ID SNR_40kHz_dB_cal
##  1: POM-1345P-C3310-R             1            67.29
##  2:       POM-2735P-R             4            65.42
##  3:    ROM-2235P-HD-R             6            82.15
##  4:    POM-2730L-HD-R            12            83.25
##  5:    AOM-5024L-HD-R            14            73.75
##  6:         ICS-40720            16           106.59
##  7:            WM-61A             7            87.53
##  8: PMM-3738-VM1000-R            21            92.22
##  9:        SPM0404UD5             9           103.42
## 10:    SPU0410LR5H-QB            20           102.52
## 11:      FG-23629-C36            17            96.75
## 12:             EM258            23            93.33
```

```
mics1[set==1,SNR_1kHz_dB_cal,.(Model,Microphone_ID)]
```

```
##                 Model Microphone_ID SNR_1kHz_dB_cal
##  1: POM-1345P-C3310-R             1           20.94
##  2:       POM-2735P-R             4           49.50
##  3:    ROM-2235P-HD-R             6           55.39
##  4:    POM-2730L-HD-R            12           60.73
##  5:    AOM-5024L-HD-R            14           76.42
##  6:         ICS-40720            16           63.93
##  7:            WM-61A             7           70.42
##  8: PMM-3738-VM1000-R            21           62.88
##  9:        SPM0404UD5             9           65.40
## 10:    SPU0410LR5H-QB            20           71.16
## 11:      FG-23629-C36            17              NA
## 12:             EM258            23           73.19
```

# Detection areas

## Draw detection spaces

prepare data with direction as angle

```
extinction.melt=melt(mics2,id=c("Code","Microphone_ID")
                     ,measure=c("extinction_distance_front_level5_1kHz_m","extinction_distance_front_40kHz_m"
                                ,"extinction_distance_left_level5_1kHz_m","extinction_distance_left_40kHz_m"
                                ,"extinction_distance_back_level5_1kHz_m","extinction_distance_back_40kHz_m"
                                ,"extinction_distance_right_level5_1kHz_m","extinction_distance_right_40kHz_m")
                     ,value.name = "Distance (m)")
```

assign angles in degrees to directions

```
extinction.melt[grepl("right",variable),c("direction","angle"):=.("right",90)]
extinction.melt[grepl("left",variable),c("direction","angle"):=.("left",270)]
extinction.melt[grepl("front",variable),c("direction","angle"):=.("front",0)]
extinction.melt[grepl("back",variable),c("direction","angle"):=.("back",180)]
```

extract frequency and estimation method

```
extinction.melt[grepl("1kHz",variable),Frequency:="1 kHz"]
extinction.melt[grepl("40kHz",variable),Frequency:="40 kHz"]
extinction.melt[grepl("extrapolated",variable),estimation:=paste("extrapolated")]
extinction.melt[is.na(estimation),estimation:="audio-visual"]
```

take average of two microphone units of one model for better graph readability

```
polar.points0=extinction.melt[,.(`Distance (m)`=mean(`Distance (m)`)),.(variable,Code,estimation,Frequency,angle,direction)]
```

add more lines to close the polar graph

```
polar.addon=polar.points0[direction=="front"]
polar.addon[,angle:=360]
polar.points1=rbind(polar.points0,polar.addon)
```

creating 2 polar plots

```
pp1=ggplot(polar.points1[Frequency=="1 kHz" & Code!="K" & estimation=="audio-visual"],aes(angle,`Distance (m)`,color=Code))+
  geom_point()+
  geom_line()+
  coord_polar()+
  ylim(c(0,max(polar.points1[,`Distance (m)`])))+
  scale_x_continuous(breaks=c(0,90,180,270))+
  scale_color_discrete(guide=F)+
  facet_wrap(~Frequency)+
  theme_cowplot()+
  background_grid(major = "xy")
```

second one with legend

```
pp2=ggplot(polar.points1[Frequency=="40 kHz"],aes(angle,`Distance (m)`,color=Code))+
  geom_point()+
  geom_line()+
  coord_polar()+
  ylim(c(0,max(polar.points1[,`Distance (m)`])))+
  scale_x_continuous(breaks=c(0,90,180,270))+
  facet_wrap(~Frequency)+
  theme_cowplot()+
  background_grid(major = "xy")
```

combining them and saving

```
plot_grid(pp1,pp2,rel_widths = c(1,1.17),ncol = 2)
```

```
ggsave("Figures/Fig S3.eps",width=8,height=5)
```

calculate detection area as sum of four quarter-ellipses

```
extinction.melt1=extinction.melt[,.(detection_area_m2=round(
  0.25*pi*`Distance (m)`[direction=="left"]*`Distance (m)`[direction=="front"]+
    0.25*pi*`Distance (m)`[direction=="front"]*`Distance (m)`[direction=="right"]+
    0.25*pi*`Distance (m)`[direction=="right"]*`Distance (m)`[direction=="back"]+
    0.25*pi*`Distance (m)`[direction=="back"]*`Distance (m)`[direction=="left"]))
  ,.(Frequency,Microphone_ID,Code,estimation)]
```

melt area and SNR data into one data table for analysis and plots

```
mics.melt0=melt(mics2,id=c("Microphone_ID","set","Code","Manufacturer")
                ,measure=c("SNR_1kHz_dB_cal","SNR_40kHz_dB_cal")
                ,value.name="SNR_dB")
```

construct labels column

```
mics.melt0[variable=="SNR_1kHz_dB_cal",Frequency:="1 kHz"]
mics.melt0[variable=="SNR_40kHz_dB_cal",Frequency:="40 kHz"]
mics.melt1=merge(extinction.melt1,mics.melt0,by=c("Microphone_ID","Frequency","Code"))
```

## Analyse relationship between SNR and detection area

Construct and test different linear models: with native SNR,
log-transformed SNR, and a curved line with polynomial relationship. We
run separate models for each frequency and estimation method because of
very different ranges and scales.

```
lm.area.1kHz=lm(detection_area_m2~SNR_dB,mics.melt1[Frequency=="1 kHz" & estimation=="audio-visual"])
lm.area.log.1kHz=lm(detection_area_m2~log(SNR_dB),mics.melt1[Frequency=="1 kHz" & estimation=="audio-visual"])
lm.area.poly.1kHz=lm(detection_area_m2~poly(SNR_dB,2),mics.melt1[Frequency=="1 kHz" & Code!="K" & estimation=="audio-visual"])

lm.area.40kHz=lm(detection_area_m2~SNR_dB,mics.melt1[Frequency=="40 kHz"])
lm.area.log.40kHz=lm(detection_area_m2~log(SNR_dB),mics.melt1[Frequency=="40 kHz"])
lm.area.poly.40kHz=lm(detection_area_m2~poly(SNR_dB,2),mics.melt1[Frequency=="40 kHz"])
```

Compare their AICcs but not R-squared values, since the polynomial model fits two coefficients.

```
AICc(lm.area.1kHz,lm.area.log.1kHz,lm.area.poly.1kHz
     ,lm.area.40kHz,lm.area.log.40kHz,lm.area.poly.40kHz)
```

```
##                    df     AICc
## lm.area.1kHz        3 367.6964
## lm.area.log.1kHz    3 362.6818
## lm.area.poly.1kHz   4 364.4879
## lm.area.40kHz       3 348.1721
## lm.area.log.40kHz   3 350.5534
## lm.area.poly.40kHz  4 349.9045
```

Linear model with log-transformation is best best for human-estimated
ranges of audible sound. Polynomial model is best for audible
extrapolated extinction distances, linear model with native SNR is best
for ultrasound. check residuals of best models

```
plot(simulateResiduals(lm.area.log.1kHz))
```

```
plot(simulateResiduals(lm.area.40kHz))
```

check results and R-squared values

```
summary(lm.area.log.1kHz)
```

```
## 
## Call:
## lm(formula = detection_area_m2 ~ log(SNR_dB), data = mics.melt1[Frequency == 
##     "1 kHz" & estimation == "audio-visual"])
## 
## Residuals:
##      Min       1Q   Median       3Q      Max 
## -1536.37  -490.68    23.11   475.75  2011.46 
## 
## Coefficients:
##             Estimate Std. Error t value Pr(>|t|)    
## (Intercept)   -26042       2255  -11.55 2.67e-10 ***
## log(SNR_dB)     8365        552   15.15 1.99e-12 ***
## ---
## Signif. codes:  0 '***' 0.001 '**' 0.01 '*' 0.05 '.' 0.1 ' ' 1
## 
## Residual standard error: 816.3 on 20 degrees of freedom
## Multiple R-squared:  0.9199, Adjusted R-squared:  0.9159 
## F-statistic: 229.7 on 1 and 20 DF,  p-value: 1.993e-12
```

```
summary(lm.area.40kHz)
```

```
## 
## Call:
## lm(formula = detection_area_m2 ~ SNR_dB, data = mics.melt1[Frequency == 
##     "40 kHz"])
## 
## Residuals:
##     Min      1Q  Median      3Q     Max 
## -1047.9  -272.9   -34.5   335.9  1590.0 
## 
## Coefficients:
##              Estimate Std. Error t value Pr(>|t|)    
## (Intercept) -4770.757    781.670  -6.103 5.78e-06 ***
## SNR_dB         78.267      9.154   8.550 4.11e-08 ***
## ---
## Signif. codes:  0 '***' 0.001 '**' 0.01 '*' 0.05 '.' 0.1 ' ' 1
## 
## Residual standard error: 587 on 20 degrees of freedom
## Multiple R-squared:  0.7852, Adjusted R-squared:  0.7745 
## F-statistic: 73.11 on 1 and 20 DF,  p-value: 4.109e-08
```

```
summary(lm.area.log.1kHz)$adj.r.squared
```

```
## [1] 0.9158823
```

```
summary(lm.area.40kHz)$adj.r.squared
```

```
## [1] 0.7744529
```

SNR has a strong, significant effect in all cases, but a poor fit with extinction distances

Construct new data table for model predictions, spanning all SNR values between extrema.

```
new.predict=data.table(SNR_dB=c(20:80,59:107)
                       ,Frequency=c(rep("1 kHz",length(20:80))
                                    ,rep("40 kHz",length(59:107))))
```

predict areas with new values

```
predict.area.1kHz0=predict(lm.area.log.1kHz,newdata = new.predict[Frequency=="1 kHz"])
predict.area.40kHz0=predict(lm.area.40kHz,newdata=new.predict[Frequency=="40 kHz"])
```

generate auxiliary data for graph

```
predict.area.1kHz1=cbind(new.predict[Frequency=="1 kHz"],area_predicted=predict.area.1kHz0)
predict.area.1kHz1[,Microphone_ID:=NA]
predict.area.40kHz1=cbind(new.predict[Frequency=="40 kHz"],area_predicted=predict.area.40kHz0)
predict.area.40kHz1[,Microphone_ID:=NA]
```

plot detection spaces against SNR

```
ggplot(mics.melt1,aes(SNR_dB,detection_area_m2,group=1))+
  geom_point(alpha=0.5)+
  # geom_text(aes(label=Microphone_ID))+
  geom_line(data=predict.area.1kHz1[area_predicted>0],aes(SNR_dB,area_predicted),size=1)+
  geom_line(data=predict.area.40kHz1[area_predicted>0],aes(SNR_dB,area_predicted),size=1)+
  labs(x= "Signal-to-noise ratio (dB, measured)",y=expression(Sound~detection~space~ground~area~(m^{2})))+
  geom_hline(yintercept = 0,lty=2)+
  facet_wrap(.~Frequency,scales = "free_x")+
  theme(legend.position = c(0.8,0.8))+
  theme_cowplot()
```

```
ggsave("Figures/Fig 2.pdf",width=8,height=5)
```

detection space increase from first usable mic to highest SNR one

```
mics.melt1[Frequency=="1 kHz" & Microphone_ID==14,.(detection_area_m2)]/mics.melt1[Frequency=="1 kHz" & Microphone_ID==4,.(detection_area_m2)]
```

```
##    detection_area_m2
## 1:          1.723474
```

```
mics.melt1[Frequency=="1 kHz" & Microphone_ID==1,.(detection_area_m2)]
```

```
##    detection_area_m2
## 1:               207
```

```
mics.melt1[Frequency=="40 kHz" & Microphone_ID==16,.(detection_area_m2)]/mics.melt1[Frequency=="40 kHz" & Microphone_ID==3,.(detection_area_m2)]
```

```
##    detection_area_m2
## 1:          10.28895
```

# Bird and bat activity

merge animal detection tags with microphones info

```
tags1=merge(mics1,tags0,by="Microphone_ID",all.x=T)
```

find most common species for choosing calls to automatically detect in next section

```
tags1[,.(activity=sum(duration_s)),.(binomial,class)]
```

```
##                   binomial    class activity
##  1:                                       NA
##  2:                     A1 MAMMALIA   114.80
##  3:                     C1 MAMMALIA  1654.71
##  4:  Dicaeum trigonostigma     AVES   997.40
##  5:       Geopelia striata     AVES   790.40
##  6:     Halcyon smyrnensis     AVES  4587.70
##  7:    Orthotomus ruficeps     AVES  5491.00
##  8:    Orthotomus sericeus     AVES   127.70
##  9:    Pycnonotus goiavier     AVES  9285.60
## 10:     Chalcophaps indica     AVES   733.30
## 11:  Pycnonotus aurigaster     AVES  1372.90
## 12:                     B1 MAMMALIA   464.80
## 13:                     D1 MAMMALIA   289.10
## 14: Orthotomus atrogularis     AVES   235.70
```

compute total activity in seconds and minutes per recording

```
tags.plot0=tags1[class!="",.(total_activity_s=round(sum(duration_s))
                             ,total_activity_min=sum(duration_s)/60)
                 ,.(Microphone_ID,recording.date,SNR_1kHz_dB_cal,SNR_40kHz_dB_cal,Code,class)]
```

manually add data for two microphones (ID 1 and 2) with no tags

```
tags.plot1=rbind(tags.plot0,cbind(mics1[Microphone_ID %in% c(1,2)
                                        ,.(Microphone_ID
                                           ,Code
                                           ,SNR_1kHz_dB_cal
                                           ,SNR_40kHz_dB_cal)]
                                  ,data.table(recording.date=c("2018-11-04","2018-11-05")
                                              ,class=c("AVES","AVES")
                                              ,total_activity_min=c(0,0)
                                              ,total_activity_s=c(0,0)))
)
```

assign SNR corresponding to frequency of vocalising animals

```
tags.plot1[class=="AVES",SNR_dB:=SNR_1kHz_dB_cal]
tags.plot1[class=="MAMMALIA",SNR_dB:=SNR_40kHz_dB_cal]
```

construct labels column

```
tags.plot1[class=="AVES",label:="Birds"]
tags.plot1[class=="MAMMALIA",label:="Bats"]
```

check distributions of bird and bat activities

Birds

```
hist(tags.plot1[label=="Birds",total_activity_s])
```

excluding outliers reveals an otherwise approximately normal distribution

```
hist(tags.plot1[label=="Birds" & Code!="A",total_activity_s])
```

Bats

```
hist(tags.plot1[label=="Bats",total_activity_s])
```

Bat activity data look right-skewed, typical for count variables. We
also have much smaller values. Bird and bat activity response variables
are on different scales (birds: minutes, bats: seconds) and distributed
differently: we need separate models again. We follow the outcome of the
previous detection area for specifying the models.

## Birds

we need to account for random effect of the date but cannot use mixed
models because there are too few levels for estimating their variance.
Activity is not a count variable per se, because it is continuous. It is
possible that it is distributed normally as it is only derived from
counts (activities are essentially weighted counts), so we need to test
different models typical for different response types. simplest case:
linear model

```
plot(simulateResiduals(lm(total_activity_min~log(SNR_dB)+recording.date,tags.plot1[label=="Birds"])))
```

the linear model behaves very well, but let’s see if a poisson glm does better

```
plot(simulateResiduals(glm(round(total_activity_min)~log(SNR_dB)+recording.date,family="poisson",tags.plot1[label=="Birds"])))
```

quantile deviations very strong. Problem may arise from overdispersion, let’s try negative binomial

```
plot(simulateResiduals(glm.nb(round(total_activity_min)~log(SNR_dB)+recording.date,tags.plot1[label=="Birds"])))
```

```
## Warning in theta.ml(Y, mu, sum(w), w, limit = control$maxit, trace =
## control$trace > : iteration limit reached

## Warning in theta.ml(Y, mu, sum(w), w, limit = control$maxit, trace =
## control$trace > : iteration limit reached
```

This did not solve the problem either.

We choose the linear model for birds because the fit is good and deviation not significant.

```
activity.model.birds=lm(total_activity_min~log(SNR_dB)+recording.date,tags.plot1[label=="Birds"])
```

check significance of model parameters as well as fits with R2 values

```
summary(activity.model.birds)
```

```
## 
## Call:
## lm(formula = total_activity_min ~ log(SNR_dB) + recording.date, 
##     data = tags.plot1[label == "Birds"])
## 
## Residuals:
##     Min      1Q  Median      3Q     Max 
## -3.5175 -0.8761 -0.5082  1.3630  3.0537 
## 
## Coefficients:
##                          Estimate Std. Error t value Pr(>|t|)    
## (Intercept)              -58.3273     5.4531 -10.696 3.14e-09 ***
## log(SNR_dB)               19.3492     1.3342  14.503 2.26e-11 ***
## recording.date2018-11-05  -3.5119     0.8613  -4.077 0.000707 ***
## ---
## Signif. codes:  0 '***' 0.001 '**' 0.01 '*' 0.05 '.' 0.1 ' ' 1
## 
## Residual standard error: 1.971 on 18 degrees of freedom
## Multiple R-squared:  0.9258, Adjusted R-squared:  0.9175 
## F-statistic: 112.2 on 2 and 18 DF,  p-value: 6.853e-11
```

```
r.squaredLR(activity.model.birds)
```

```
## [1] 0.9257573
## attr(,"adj.r.squared")
## [1] 0.9269677
```

SNR explains a lot of variation for bird activity

Generate new data tables for predicting values and plotting.

```
new.data.birds=data.table(tags.plot1[label=="Birds",.(SNR_dB=seq(min(SNR_dB),max(SNR_dB),0.5))]
                          ,tags.plot1[label=="Birds",.(recording.date=unique(recording.date))])
```

```
## Warning in as.data.table.list(x, keep.rownames = keep.rownames, check.names
## = check.names, : Item 2 has 2 rows but longest item has 111; recycled with
## remainder.
```

predict values with chosen model

```
predict.activity.birds=data.table(total_activity_min_predicted=predict(activity.model.birds,newdata=new.data.birds),new.data.birds,label="Birds")
```

## Bats

Bat activities, because of the right skew, behave more like a count
variable. We use the interaction between day and SNR this time, because
we observed strong differences between nights that might cause slightly
different relationships.

generalised linear model for count variable (poisson)

```
plot(simulateResiduals(glm(total_activity_s~SNR_dB*recording.date,family="poisson",tags.plot1[label=="Bats"])))
```

Too many outliers, problem may arise from overdispersion, let’s try negative binomial

```
plot(simulateResiduals(glm.nb(total_activity_s~SNR_dB*recording.date,tags.plot1[label=="Bats"])))
```

fixes some problems. Gamma might be better because we have a continuous response variable.

```
plot(simulateResiduals(glm(total_activity_s~SNR_dB*recording.date,family=Gamma(link="log"),tags.plot1[label=="Bats"])))
```

We choose this as the best model fit so far.

```
activity.model.bats=glm(total_activity_s~SNR_dB*recording.date,family=Gamma(link="log"),tags.plot1[label=="Bats"])
```

check significance of model parameters as well as fits with R2 values

```
summary(activity.model.bats)
```

```
## 
## Call:
## glm(formula = total_activity_s ~ SNR_dB * recording.date, family = Gamma(link = "log"), 
##     data = tags.plot1[label == "Bats"])
## 
## Deviance Residuals: 
##      Min        1Q    Median        3Q       Max  
## -0.97667  -0.32768  -0.01821   0.26049   0.68977  
## 
## Coefficients:
##                                  Estimate Std. Error t value Pr(>|t|)    
## (Intercept)                     -6.166242   0.881932  -6.992 8.75e-07 ***
## SNR_dB                           0.108950   0.009926  10.976 6.46e-10 ***
## recording.date2018-11-04         6.079401   1.229888   4.943 7.84e-05 ***
## SNR_dB:recording.date2018-11-04 -0.049330   0.014317  -3.446  0.00256 ** 
## ---
## Signif. codes:  0 '***' 0.001 '**' 0.01 '*' 0.05 '.' 0.1 ' ' 1
## 
## (Dispersion parameter for Gamma family taken to be 0.2084408)
## 
##     Null deviance: 30.9526  on 23  degrees of freedom
## Residual deviance:  4.6062  on 20  degrees of freedom
## AIC: 231.77
## 
## Number of Fisher Scoring iterations: 9
```

```
r.squaredLR(activity.model.bats)
```

```
## [1] 0.8749641
## attr(,"adj.r.squared")
## [1] 0.8749747
```

SNR explains a lot of variation for bat activity

Generate new data tables for predicting values and plotting.

```
new.data.bats=data.table(tags.plot1[label=="Bats",.(SNR_dB=seq(min(SNR_dB),max(SNR_dB),0.5))]
                         ,tags.plot1[label=="Bats",.(recording.date=unique(recording.date))])
```

```
## Warning in as.data.table.list(x, keep.rownames = keep.rownames, check.names
## = check.names, : Item 2 has 2 rows but longest item has 95; recycled with
## remainder.
```

predict values with chosen model

```
predict.activity.bats=data.table(total_activity_min_predicted=exp(predict(activity.model.bats,newdata=new.data.bats))/60,new.data.bats,label="Bats")
```

plot activity data and model predictions

```
ggplot(tags.plot1,aes(SNR_dB,total_activity_min,color=recording.date,group=recording.date))+
  geom_hline(yintercept=0,lty=2)+
  geom_point(alpha=0.5)+
  # geom_text(aes(label=Microphone_ID))+
  geom_line(data=predict.activity.birds[total_activity_min_predicted>0],aes(SNR_dB,total_activity_min_predicted),size=1)+
  geom_line(data=predict.activity.bats,aes(SNR_dB,total_activity_min_predicted),size=1)+
  facet_wrap(.~label,scales="free")+
  scale_color_discrete(name="Date")+
  labs(x="Signal-to-noise ratio (dB, measured)",y="Total vocalisation activity (min)")+
  theme_cowplot()+
  theme(legend.position = c(0.05,0.8))
```

```
ggsave("Figures/Fig 3.pdf",width=8,height=5)
```

Bird activity increase from first usable mic to highest SNR one, for most active day

```
tags.plot1[label=="Birds" & Microphone_ID==14,.(total_activity_s)]/tags.plot1[label=="Birds" & Microphone_ID==4,.(total_activity_s)]
```

```
##    total_activity_s
## 1:             1.62
```

likewise, we compute the bat activity increase

```
tags.plot1[label=="Bats" & Microphone_ID==19,.(total_activity_s)]/tags.plot1[label=="Bats" & Microphone_ID==3,.(total_activity_s)]
```

```
##    total_activity_s
## 1:          9.69697
```

# Bird and bat species accumulation curves

number of time steps over which to calculate species accumulation curves

```
n_timesteps=40
```

duration of recordings in seconds

```
total_duration_birds=900
total_duration_bats=900
```

add duration of first ultrasound recording to tag time of second recording to simulate one recording

```
tags1[class=="MAMMALIA" & grepl("_181500",recording.name),c("min_time_adjusted","max_time_adjusted"):=.(min_time+(30*60),max_time+(30*60))]
```

initiate empty data table and run loop for counting species at each time step for each microphone and taxon

```
richness.cumulative0=data.table()
for (c in unique(tags1[class!="",class])){
  if (c=="AVES") {total_duration=total_duration_birds}
  if (c=="MAMMALIA") {total_duration=total_duration_bats}
  for (t in 1:n_timesteps){
    end=total_duration*(t)/n_timesteps
    #'count species
    richness.cumulative.temp=tags1[class==c,.(richness=length(unique(binomial[min_time<end]))
                                              ,class=unique(class))
                                   ,.(Microphone_ID,SNR_1kHz_dB_cal,SNR_40kHz_dB_cal,recording.date)]
    #'append results into the data table
    richness.cumulative0=rbind(richness.cumulative0
                               ,cbind(richness.cumulative.temp,time=end,time_bin=t))
  }
}
```

append missing data from 2 microphones that did not record birds manually

```
richness.cumulative1=rbind(richness.cumulative0
                           ,data.table(Microphone_ID=c(1,2)
                                       ,class=c("AVES","AVES")
                                       ,mics1[Microphone_ID %in% c(1,2),.(SNR_1kHz_dB_cal)]
                                       ,recording.date=c("2018-11-04","2018-11-05")
                                       ,richness=0
                                       ,time=rep(richness.cumulative0[,unique(time)],each=2))
                           ,fill=T)
```

fill in time bins for missing data

```
richness.cumulative1[is.na(time_bin) & class=="MAMMALIA",time_bin:=(time*n_timesteps)/total_duration_bats]
richness.cumulative1[is.na(time_bin) & class=="AVES",time_bin:=(time*n_timesteps)/total_duration_birds]
```

put SNR into one column

```
richness.cumulative1[class=="AVES",SNR:=SNR_1kHz_dB_cal]
richness.cumulative1[class=="MAMMALIA",SNR:=SNR_40kHz_dB_cal]
```

create labels column

```
richness.cumulative1[class=="AVES",label:="Birds"]
richness.cumulative1[class=="MAMMALIA",label:="Bats"]
```

remove duplicates to shorten

```
richness.cumulative_notime=copy(richness.cumulative1)
richness.cumulative_notime[,c("time_bin","time"):=.(NULL,NULL)]
richness.cumulative2=richness.cumulative1[!duplicated(richness.cumulative_notime) | time_bin==1 | time_bin==n_timesteps]
```

generate two graphs separately (for maximal separation between color gradient extremes)

```
pcum1=ggplot(richness.cumulative2[class=="MAMMALIA"],aes(time+1,richness,group=Microphone_ID,color=SNR))+
  geom_line(size=1.5,color="black")+
  geom_line(size=1)+
  scale_color_gradient(low="white",high="black",name="Signal-to-noise ratio \n@40 kHz (dB)")+
  labs(x=NULL,y="Bat species richness")+
  facet_wrap(.~recording.date,scales="free")+
  theme_cowplot()+
  theme(panel.grid.major.y=element_line(linetype=3,colour="darkgrey"))
pcum2=ggplot(richness.cumulative2[class=="AVES"],aes(time+1,richness,group=Microphone_ID,color=SNR))+
  geom_line(size=1.5,color="black")+
  geom_line(size=1)+
  scale_color_gradient(low="white",high="black",name="Signal-to-noise ratio \n@1 kHz (dB)")+
  labs(x="Sampling time (s)",y="Bird species richness")+
  facet_wrap(.~recording.date,scales="free")+
  theme_cowplot()+
  theme(panel.grid.major.y=element_line(linetype=3,colour="darkgrey"))
plot_grid(pcum1,pcum2,ncol=1)
```

```
ggsave("Figures/Fig 4.eps",width=9,height=8)
```

# Automated call detection

list files in subdirectory “call detection”

```
files=data.table(filepath=list.files(path="call detection",pattern=".wav",full.names = T)
                 ,filename=list.files(path="call detection",pattern=".wav"))
```

distingish templates from soundscapes

```
files[grepl("call",filename),template:=T]
files[is.na(template),template:=F]
```

extract microphone ID from file name

```
files[,Microphone_ID:=as.numeric(substr(filename,1,2))]
```

extract time of day info

```
files[grepl("180000",filename),daytime:="night"]
files[is.na(daytime),daytime:="day"]
files[,mic_daytime:=paste(Microphone_ID,daytime)]
files[template==F,date:=tstrsplit(filename,"_")[3]]
```

Only run lengthy automated detection if the file has not been generated yet by a previous script run.

```
if (!file.exists("Detected calls.csv")){
  # create recipient data table for all detections
  detections.all=data.table()
  # run loop over all microphone-daytime combinations
  # for (md in files[,unique(mic_daytime)]){
  for (md in "24 day"){
    # run loop over all microphone templates for each microphone
    r=files[mic_daytime==md,unique(Microphone_ID)]
    #extract date
    date_temp=files[template==F & mic_daytime==md,date]
    #inform loop progress
    print(paste(md,date_temp))
    # set the frequency range of the signal to detect
    if (grepl("night",md)) {freq_temp=c(35,80)
    name_temp="bat A";d="night";window_temp=256;f_range=c(30,90)
    } else if (grepl("day",md)) {freq_temp=c(1,3.5)
    name_temp="bulbul";d="day";window_temp=128;f_range=c(0,4)}
    # extract call count from microphones data table
    if (d=="night") {call_count=mics1[Microphone_ID==r,BatA_counted_calls]
    } else if (d=="day") {call_count=mics1[Microphone_ID==r,Bulbul_counted_calls]}
    
    # build first and second call template
    temp1=makeCorTemplate(files[template==T & Microphone_ID==r & daytime==d,filepath][1],frq.lim=freq_temp,wl=window_temp,name=paste(name_temp,1))
    temp2=makeCorTemplate(files[template==T & Microphone_ID==r & daytime==d,filepath][2],frq.lim=freq_temp,wl=window_temp,name=paste(name_temp,2))
    # scan recording with template
    soundscape.temp=files[template==F & mic_daytime==md,filepath]
    matches.temp1=corMatch(soundscape.temp,temp1,time.source = "fileinfo")
    matches.temp2=corMatch(soundscape.temp,temp2,time.source = "fileinfo")
    # take only the peaks from the detection timeline with a minimal cutoff
    templateCutoff(matches.temp1)=c(default=0.05)
    templateCutoff(matches.temp2)=c(default=0.05)
    peaks1=findPeaks(matches.temp1)
    peaks2=findPeaks(matches.temp2)
    
    detections0=data.table()
      for (t in c(1,2)){
        #take one of the two peak objects corresponding to the template
        if (t==1) {peaks=peaks1} else if (t==2) {peaks=peaks2}
        detections.temp0=data.table(peaks@detections[[1]])
      #create id column
        detections.temp0[,id:=1:.N]
      # order detections by score to work our way down
      detections.temp1=detections.temp0[order(-score)]
      for (i in 1:nrow(detections.temp1)) {
        # verify all peaks manually
        print(paste("detection:",detections.temp1[i,id],"template:",t,". Enter y if detection is correct, n if not"))
        verify_temp=readline(showPeaks(peaks,flim=f_range,id=detections.temp1[i,id]))
        detections.temp1[i,true:=verify_temp]
        # stop if ten consecutive detections are false positives
        if (verify_temp=="n"){consecutive_false_positives=consecutive_false_positives+1
        } else if (verify_temp=="y") {consecutive_false_positives=0}
        if (nrow(detections.temp1[true=="y"])==call_count | consecutive_false_positives==20){detections.temp1[is.na(true),true:="n"];break}
      }
      #assign microphone, daytime, and template info
      detections.temp1[,template:=t]
      detections0=rbind(detections0,detections.temp1)
      }
      detections0[,mic_daytime:=md]
    # append output (in case of crash)
    fwrite(detections0,"Detected calls.csv",append=T)
    # and save in object too
    detections.all=rbind(detections.all,detections0)
  }
} else {
  # this imports the file consisting of detection results
  detections.all=fread("Detected calls.csv")}
```

Compute the number of true and false positives at different score cutoffs.

```
auto.detect0=data.table()
for (s in seq(1,0.05,-0.01)){
  for (md in detections.all[,unique(mic_daytime)]) {
    #take average number of positives per template
    true_positives=nrow(detections.all[score>=s & true=="y" & mic_daytime==md])/2
    false_positives=nrow(detections.all[score>=s & true=="n" & mic_daytime==md])/2
    # store in data table
    auto.detect0=rbind(auto.detect0,data.table(true_positives,false_positives,score_cutoff=s,mic_daytime=md))
  }
}
```

extract day time, taxon, and microphone ID

```
auto.detect0[,Soundscape_microphone_ID:=as.numeric(unlist(tstrsplit(mic_daytime," ")[1]))]
auto.detect0[,daytime:=tstrsplit(mic_daytime," ")[2]]
auto.detect0[daytime=="day",Label_calls:="Bird calls"]
auto.detect0[daytime=="night",Label_calls:="Bat calls"]
```

merge automated detections data with microphones data

```
auto.detect1=merge(auto.detect0,mics1[,.(Microphone_ID,BatA_counted_calls,Bulbul_counted_calls,SNR_40kHz_dB_cal,SNR_1kHz_dB_cal,set)]
                   ,by.x="Soundscape_microphone_ID",by.y="Microphone_ID")
```

put manually counted calls total in one column

```
auto.detect1[daytime=="night",counted_calls:=BatA_counted_calls]
auto.detect1[daytime=="day",counted_calls:=Bulbul_counted_calls]
```

define set labels

```
auto.detect1[set==1,set_label:="Set 1"]
auto.detect1[set==2,set_label:="Set 2"]
```

compute recall and precision, as well as total detections

```
auto.detect1[,recall:=true_positives/counted_calls]
auto.detect1[,total_detections:=true_positives+false_positives]
auto.detect1[,precision:=true_positives/(true_positives+false_positives)]
```

assign SNR values depending on time of day

```
auto.detect1[daytime=="night",SNR_dB:=SNR_40kHz_dB_cal]
auto.detect1[daytime=="day",SNR_dB:=SNR_1kHz_dB_cal]
```

calculate the maximal score for an acceptable recall of 0.5, and the minimum score for an acceptable precision of 0.5

```
acceptable=auto.detect1[,.(recall_max_score=max(score_cutoff[recall>=0.5])
                           ,precision_min_score=min(score_cutoff[precision>=0.5],na.rm=T))
                        ,.(mic_daytime,Soundscape_microphone_ID,daytime,SNR_dB,set_label,Label_calls)]
```

```
## Warning in max(score_cutoff[recall >= 0.5]): no non-missing arguments to max;
## returning -Inf

## Warning in max(score_cutoff[recall >= 0.5]): no non-missing arguments to max;
## returning -Inf

## Warning in max(score_cutoff[recall >= 0.5]): no non-missing arguments to max;
## returning -Inf

## Warning in max(score_cutoff[recall >= 0.5]): no non-missing arguments to max;
## returning -Inf

## Warning in max(score_cutoff[recall >= 0.5]): no non-missing arguments to max;
## returning -Inf
```

some values have to be replaced with 0

```
acceptable[recall_max_score<0,recall_max_score:=0]
acceptable[recall_max_score>precision_min_score,range_acceptable:=recall_max_score-precision_min_score]
acceptable[recall_max_score<=precision_min_score,range_acceptable:=0]
```

melt data for graphing

```
acceptable.melt=melt(acceptable,id=c("mic_daytime","Soundscape_microphone_ID","daytime","SNR_dB","Label_calls","set_label"),measure=c("recall_max_score","precision_min_score"))
auto.detect.melt=melt(auto.detect1,id=c("SNR_dB","score_cutoff","set_label","daytime","Label_calls"),measure=c("total_detections","false_positives","counted_calls"),value.name="Calls/detections")
```

```
## Warning in melt.data.table(auto.detect1, id = c("SNR_dB", "score_cutoff", :
## 'measure.vars' [total_detections, false_positives, counted_calls] are not all
## of the same type. By order of hierarchy, the molten data value column will be of
## type 'double'. All measure variables not of type 'double' will be coerced too.
## Check DETAILS in ?melt.data.table for more on coercion.
```

define labels

```
auto.detect.melt[variable=="counted_calls",variable:="Total calls"]
auto.detect.melt[variable=="total_detections",variable:="True positives"]
auto.detect.melt[variable=="false_positives",variable:="False positives"]
auto.detect.melt[`Calls/detections`==0,`Calls/detections`:=NA]
auto.detect.melt.ordered=auto.detect.melt[order(variable)]
```

plot total calls, true positives, false positives, and levels of acceptable recall and precision

```
ggplot(auto.detect.melt.ordered,aes(SNR_dB,score_cutoff))+
  # geom_ribbon(data=acceptable,aes(SNR_dB,ymax=recall_max_score,ymin=precision_min_score,y=1),color="green",alpha=0.3)+
  geom_point(aes(size=`Calls/detections`,color=variable),shape=15)+
  scale_size_continuous(range=c(0.5,10))+
  # geom_line(data=acceptable.melt,aes(SNR_dB,value,lty=variable),color="green",size=0.8)+
  geom_errorbar(data=acceptable[range_acceptable>0],aes(SNR_dB,ymax=recall_max_score,ymin=precision_min_score,y=1),color="green",size=1,width=1)+
  scale_color_manual(values = c("black","blue","red"),name="")+
  facet_grid(set_label~Label_calls,scales="free")+
  theme_cowplot()+
  background_grid(major="y")+
  labs(y="Score cutoff",x="Signal-to-noise (dB)")
```

```
## Warning: Removed 4008 rows containing missing values (geom_point).
```

```
ggsave("Figures/Fig 5.eps",width=10,height=8)
```

```
## Warning: Removed 4008 rows containing missing values (geom_point).
```

test effect of SNR on range of acceptable scores

```
acceptable.model=lm(range_acceptable~SNR_dB*daytime+set_label,acceptable)
plot(simulateResiduals(acceptable.model))
```

```
summary(acceptable.model)
```

```
## 
## Call:
## lm(formula = range_acceptable ~ SNR_dB * daytime + set_label, 
##     data = acceptable)
## 
## Residuals:
##       Min        1Q    Median        3Q       Max 
## -0.094678 -0.046344 -0.001832  0.044198  0.167510 
## 
## Coefficients:
##                      Estimate Std. Error t value Pr(>|t|)    
## (Intercept)         -0.333954   0.121779  -2.742 0.009549 ** 
## SNR_dB               0.009306   0.001859   5.006 1.58e-05 ***
## daytimenight         0.044555   0.160917   0.277 0.783497    
## set_labelSet 2      -0.071129   0.019591  -3.631 0.000895 ***
## SNR_dB:daytimenight -0.005017   0.002206  -2.274 0.029197 *  
## ---
## Signif. codes:  0 '***' 0.001 '**' 0.01 '*' 0.05 '.' 0.1 ' ' 1
## 
## Residual standard error: 0.06131 on 35 degrees of freedom
## Multiple R-squared:  0.789,  Adjusted R-squared:  0.7649 
## F-statistic: 32.73 on 4 and 35 DF,  p-value: 2.207e-11
```

```
summary(acceptable.model)$adj.r.squared
```

```
## [1] 0.7649346
```

explore interaction of SNR and daytime

```
test(emtrends(acceptable.model,"daytime",var="SNR_dB"))
```

```
##  daytime SNR_dB.trend      SE df t.ratio p.value
##  day          0.00931 0.00186 35 5.006   <.0001 
##  night        0.00429 0.00119 35 3.613   0.0009 
## 
## Results are averaged over the levels of: set_label
```
